# Abstract of the Introductory Address Delivered at the Buffalo Medical College, Wednesday, Nov. 3d, 1875

**Published:** 1875-11

**Authors:** E. V. Stoddard


BUFFALO

### Medical and Surgical Journal.

VOL XV. NOVEMBER, 1875. No. 4.

### Original Communications.

:o:

ART. 1.—*Abstract of the Introductory Address delivered at the Buffalo Medical College, Wednesday Evening, Nov.* 3, 1875. By Prof. E. V. STODDARD, M. D.

GENTLEMEN:—The pleasant duty of welcoming another class to the Buffalo Medical College has devolved upon me.

To those who have for the first time entered these halls, as students, as well as to those who have, at a previous session, filled these seats, in behalf of the Faculty, I extend a cordial welcome, coupled with the hope that the present may prove the commencement of a long and prosperous career,

You expect at this time some words of counsel, some practical suggestions, which not only may stand as guides in your labors here, but shall serve as a basis of action in the future as well. It has seemed to me that, in any work, a full understanding of its character and bearings is necessary to success in its pursuit; and influenced by this view, I have thought it best to invite your attention to the character of the Medical Science of to-day, its past and present relations, and your own relations to the studies in which you are engaged.

There are periods in the experience of the individual when it is well for him to review his progress, to consider his successes and his failures in the past, and his present relation to his surroundings. What is true of the individual is also true of a profession. And it is well that, at times, its members look back over its past, and study carefully its present status. To those about entering it, such a review is of special importance and value, for its two great problems of absorbing interest of to-day are to know what point we have reached, and whither we are tending. * *

One of the greatest hindrances to the progress of our science has been a blind deference to so-called *authority*. In its early history, when knowledge was confined to a comparatively limited number, this was almost inevitable; but even in more modern times this same spirit has prevailed. For nearly thirteen centuries the teachers of anatomy propagated the error, taught by Galen, that the blood passed from one ventricle to the other through small openings, until Vesalius ventured to question the truth of the long venerated authority, and demonstrated that no such openings exist, and this made one of the steps in advance which enabled Harvey later to demonstrate the circulation of the blood.

The *character* of the work before you, gentlemen, is *Scientific*, because it seeks to attain knowledge in the development of facts.

All sciences are included under two divisions, the *exact* and *inexact*. The *exact* comprising those whose results are susceptible of absolute demonstration, such are Mathematics and Logic. The *inexact* are those whose results are not capable of absolute demonstration, but are subject to certain conditions which modify the exactness of the interpretation of their phenomena.

A science, to be exact, requires the completed and classified observation of all the facts or phenomena which can possibly come within its scope. An inexact science, on the contrary, is one the phenomena of which, from its extent and relations to other sciences, can never be fully observed and classified, and hence its general laws cannot be capable of absolute demonstration.

Medicine belongs to the inexact sciences, since it is characterized by the same features.

Its observed phenomena are not all referable to principles which are capable of positive demonstration. It is one of the natural sciences. Its field of observation is *nature*, and the complexity of its relations precludes the possibility of observing, in a limited period, *all* its phenomena.

The facts of medical science which have been recognized and recorded, form but a part only of those which may come within its bounds, and from a consideration of these facts the laws deduced can only be relative and not absolute.

Among the inexact sciences we meet different degrees of uncertainty; this arises from two causes; *first*, inaccurate observations of facts, and, *second*, too limited a number of facts upon which scientific generalizations are based. * * * * *

This rigid mode of examination is too often wanting, and phenomena imperfectly observed are offered as facts upon which to base deductions of general truth.

Again, when the relations of a science are so extended and complex, the number of facts from which general laws are to be constructed must be very extensive, and, to obtain such a number, a long series of observations is necessary. * * * *

It is a truth which we are compelled to acknowledge, that in all the departments of medical science there has been, to a greater or less extent, a failure to recognize these cardinal points of scientific method. Many of the phenomena of medical science have been hastily considered, and imperfect and erroneous views of them announced. From the grouping together of a limited number of such phenomena, hasty generalizations have been made which have been announced as established laws. Hence there has arisen in the minds of many a *skepticism* in regard to medicine that would deprive it of its character as a science.

You may ask why consider this skeptical tendency important, since medicine is too firmly established as a science to be influenced by the skeptical indifference of a few of its followers? The existence of any erroneous tendency of thought in science is a grave matter. Evils grow; and in an inexact science, many of whose phenomena are inexplicable, such skepticism calls for serious consideration. * * * * * * * * *

Nor is this skepticism confined to medicine alone; it is the tendency of the times. There exists a habit of thought, vigorous, bold and iconoclastic, which has pervaded almost every department of human knowledge. It has overturned theories and systems which had been considered fixed and immovable. * *

Recognizing the existence of this spirit of rigid investigation, we are led to understand the real causes of this skepticism.

They are twofold; *first*, the difficulty of observing the phenomena of medical science from their complexity; *second*, the tendency to hasty generalization from small groups of facts. * *

This error is not the *least* common into which medical philosophy has fallen. Observers in medical science, losing sight of the fact that medicine differs from mathematics in exactness, from a few imperfectly group phenomena, boldly announce a law, which, when tested by experience falls far short of explaining what it claims. This tendency to apply deductive reasoning to facts of medical science, has ever proved a prolific source of error. The assumption of a complete general law from a few incomplete generalizations of facts, has operated more decidedly to shake confidence in, and confuse the principle of medicine, than any other source of error which has prevailed.

The mathematician, dealing with absolute and exact facts, announces his general law. From this general law, by a systematic chain of deductions, he proceeds to the consideration of individual facts. His general law being perfect and universal, the conclusions reached under it, in regard to his individual facts, must be accurate and complete also. He thus reasons from the general to the particular, and his deductions as to the individual fact are as accurate and perfect as the general law from which his process of reasoning started.

Such is not the case with the medical philosopher. He is the observer of individual or scattered phenomena having differing relations and tendencies.

Starting with his individual facts, or group of facts, he seeks to frame some general principle or law which shall include such phenomena as he may have observed.

He thus proceeds from the particular to the general; or reasons, in other words, by an inductive process, from the facts observed to generalizations based upon these facts. Herein lies the danger. The number of his facts collected being limited and not including all the phenomena which may appear in this direction, the generalizations drawn from them are not sufficiently comprehensive, and fail to explain the phenomena observed by others when tested by this law.

The failure of the laws of the science in any part to explain the phenomena arising under such laws necessarily generates a want of confidence in such laws, and the transition from partial distrust to complete skepticism is rapid. Thus we readily recognize the second of the two prominent causes of the two skeptical tendency which all have been considering.

With the evil before us it is our province to seek for and apply its remedy, and the nature of the remedy is at once suggested.

In *systematic method* applied to observation and deduction we find a ready and efficient means of combating the sceptical tendency to which I have referred.

This faulty and unscientific generalization produces skepticism by the substitution for facts, of processes of reasoning or theories based upon unsufficient premises. Encouraging false opinions, hindering scientific investigation it has produced confusion between correct and incorrect theory.

You may ask why, if theories in medicine be so apt to mislead, it may not be better to discard theory and trust wholly to observation and experience, as did the Empiric school of an early day. I readily admit that a false theory is worse than no theory at all. But pure empiricism is as impossible as pure rationalism.

Every one of us has our theory of disease and treatment. We now and then meet one who will maintain that he has no theories but is entirely *practical.* Such a man *has* theories nevertheless, and generally founded upon a very unsubstantial basis, inasmuch as he is in reality an unconscious theorist who is unable to distinguish between his own imaginings and real phenomena.

Theory is a necessity in all science, and a sound and well grounded theory is not only the means of connection between numerous complex phenomena, but the key by which the intricacies of the problem may be unlocked.

The last half century has been specially marked by a growing spirit of earnest inquiry. It has not been sudden in its commencement. We trace it back to its origin in the beginning of the 16th century, when with the birth of Protestantism, a tendency to liberality of opinion and seeking for the truth was developed. This has steadily increased with the diffusion of knowledge till, little by little, the web of error, which was interwoven with a warp of truth, has been unravelled and a purer fabric of facts has been formed as the mantle for science.

This healthier state of inquiry has changed the aspect of many of the physical and organic sciences; has overturned beliefs once considered unmovable, and has caused a distrust of and want of confidence in others which were considered equally secure. To such an extent has this spirit developed, that in no period of the history of medical science has existed so healthy a state of observation. Let but a new fact be announced in any department, and a hundred observers immediately turn all their powers of analysis to test its accuracy and truth. How different this condition from that of the earlier history of our sciences!

Let me for a moment briefly review some of the prominent eras in the past of medicine in illustration.

From the period when medicine ceased to be purely an art, and assumed the character of a science, it has been influenced by all the prevailing tendencies of human thought.

Perhaps the most marked and tenacious error of the past has been the reverence for *authority*. I have already referred to the influence which this blind adherence to opinion has exerted.

This was broken in upon by the period of charlatanry, and faith in specifics which followed it, and prepared the way for the introduction of metaphysical systems to interpret and explain the nature of the phenomena of health and disease.

The confusion of truth and error in medical science was correspondent with that which existed in the so called schools of philosophy, which were the outgrowth of the idea then prevalent that logic and philosophy were the crowning studies of the human intellect. * * * * * *

*Again*, the *belief in specific remedies* which led, in this earlier day, to the long and unavailing search for the "*Elixir vitæ*" and "*Philosopher's stone*," has been specially hostile to the advance of science. * * * * * * *

*Another* prominent error of the past has been a *speculative pathology* or an inadequate recognition and interpretation of the causes of disease. Upon the prevailing theories of the causation of disease have been built, systems of therapeutics which have resulted most disastrously.

The error so long prevalent, that disease is something *added to* the body, possessing an individual and distinct existence is passing away, and instead, the fact, that it is a perverted physiological process or function, a lessened vital power, has taken its place, and has developed what Dr. Chambers has so aptly denominated "Restorative medicine."

These brief illustrations of the errors of the past, and the tendency of the present to a truer and more rational medicine, are sufficient to show the influence of the earnest and scientific spirit of investigation, which I have described.

They show us that the period of unthinking belief, and speculation has passed away, that, commencing in a simple empricism, medicine has by successive stages grown into a *comparatively* positive science. The facts accumulated by the observations of the past have been carefully analyzed and gathered into a form which constitutes positive knowledge. And in the application of this knowledge we recognize the great developement and progress of the present.

This we will consider under the departments of *preventive* and *curative* medicines.

Preventive medicine manifests its power in a two fold manner; *first*, by modifying existing disease; and *secondly*, by preventing its development; both of these characteristics are based upon a better and more perfect knowledge of the predisposing and exciting causes of disease. * * * * * *

In *curative* medicine the advances have been no less signal than in preventive medicine.

A more accurate pathology and physiology have given us a more scientific therapeutics; not only have sounder views of rational treatment of special diseases, based on advances in pathological knowledge, been adopted, but the Pharmacopœia has been enriched by invaluable additions derived from a better knowledge of the physiological action of remedies.

From the more accurate pathology of to-day, how opposite to that formerly followed is the treatment of the inflammatory and febrile diseases. The recognition of the tendency to self limitation of disease after the completion of its characteristic cycle, has abundantly proven that the restorative treatment of to-day offers far better results than the depleting and abortive practice of the past.

Another advance in curative medicine has arisen from the assistance derived in special diseases from the use of special instruments employed to detect textural changes and morbid processes, which before were not recognized.

Another great advance in modern medicine has been the result of a diffusion of a better and more correct understanding of the word *cure*, and of the distinction between the idea of curing the disease and curing the patient.

We seek the cause and remove it, while nature completes the cure by removing the visible symptoms.

In this brief survey of the past and present of medical science, I have endeavored to point out its characters as a science, the prominent errors into which it has fallen, and by a brief analysis of their causes to show its present condition and tendencies.

In considering the relations of medicine, past and present, one cannot fail to be impressed with its dependence upon the character of any period of its history which we may review.

The progress of all science is parallel with and accompanies the development of civilization; medical science shares in this process of development, and so perfectly does it reflect the prevailing tendency of thought, in any age of man's history, that the condition of medical science may be taken as the measure of civilization of that period.

The relations of medicine to science to-day, are both direct and indirect.

In its *direct* relations, it follows the progress of science, adopting its conclusions and appropriating its advances to the development of its own departments.

The more fully developed laws of light, heat, sound, electricity, and of chemistry, have directly contributed to the expansion of medical science, rendering physiology more perfect and physical inquiry into disease more exact and certain.

In its *indirect* relations to physical science, medicine offers even more decided evidence of its dependence upon a parallel development. I have already referred to the influence of the *temper* of modern science upon modern medicines, in the production of a skeptical tendency of thought in observation. In a period of such rapid advancement as the present, careful scrutiny of proffered opinions is indispensable, we must, however, guard against too hastily discarding the accumulated experiences of those who have preceded us. In the empiricism of the past we may find a store of practical wisdom.

We are undoubtedly in advancee of our fathers, but we must not lose sight of the fact, that with the gains in ease and perfection of observation, we are liable to losses in being deprived of that patient study of detail and reserve of statement which less facile means of research have given those who have preceded us.

The *temper* of modern science has other relations to medicine than that to which I have just referred; and what this temper is, it will be well for a moment to consider. Dr. Acland, in referring to it, says; "It would be difficult more aptly to describe it than by the words of Newton: 'The main business of natural philosophy is to argue from phenomena without feigning hypotheses, and deduce causes from effects, till we come to the very *first cause,* which certainly is not mechanical.' To discuss this simple phrase and expand it to its full significance, would be to recapitulate the history of a great part of modern science, There probably is no part to which some modern thinkers would not take exception. But it cannot fail to raise, in every mind, a splendid and affecting image of the boundless field of physical philosophy. It will suggest to one a countless host of loving worshippers; to another, it reveals a crowd of stern enquirers ardently groping in dim, cold twilight. Each in his own sphere, each tinged with a special hue of his own nature in Physics and Biology, all alike are searching for a *true cause*."

What ends have they attained? The statement that all action of which we are conscious is the result of solar heat upon interdependent and correlative existences. That, in this system, things are capable only of interchange; that nothing now existing is destroyed; that no new energy is created.

Does this theory of correlation of forces bear upon medical science? Indirectly we trace its effects.

The belief which so long prevailed that a vital principle was the controlling force of organic life, is now abandoned.

The hypothesis of Darwin is rendered worthy of but little attention since, by this law of correlation, it is shown that mental correlations extend into all structure and growth, and that living beings, placed in similar conditions, will proceed in parallel lines, and conversely also.

These ideas, at first sight, seem to have but little bearing upon an art which has to do entirely with the work of every-day life. Yet we see, from a moment's reflection, that a pursuit devoted to the study of the natural sciences must be indirectly, as well as directly, influenced by the general temper and character of scientific thought.

But we must pass to another of its relations, that is, its relation to the wants of man in the complications of society.

Your attention has been called to this question at some length during the past four weeks, as you have followed me in the discussion of the topics of municipal Hygiene. The present day has made *public* and *preventive* medicine a great branch of medical science.

The social questions of the time, the problems of political economy, all bear upon and mingle with the studies which sanitary science offers.

I will not weary you with a further discussion of these relations of the medicine of to-day, but pass to the consideration of one or two topics which the occasion would render specially significant.

If the relations of medicine be such as I have indicated, *what should be the character of the preparation* of the physician for his duties. It is certain that he should be many sided, that his powers of observation should be thoroughly schooled and trained to precision and exactness in perception and thought.

This is to be accomplished by a *liberal education.*

And here I know that I tread upon debatable ground. Two rival methods claim our attention, the *Classical* and the *Scientific.* In no partisan spirit, we can say *both* are equally of value.

From the Scientific we get that intellectual culture which gives strength and massiveness. From the Classical is derived the æsthetic element of culture which develops the emotional part of our nature.

To separate the moral and emotional from the intellectual in culture is an impossibility. So, in a conception of culture, liberal in character, the separation of the scientific and the classical is impossible. It is by the union of these two means that we achieve what I would designate as a *liberal education.*

We cannot, then, in such an education, be bound to a rigid and scientific method alone, but must cultivate the the social portions of our nature by a blending of the æsthetic with the scientific.

Professor Tyndall, in a recent address, in discussing the culture of the scientific man, says: "Man is not all intellect. If he were so, science would be his proper nutriment. He *feels* as well as *thinks*, he is receptive of the sublime and beautiful as well as of the true.

"The circle of human nature, then, is not complete without the arc of feeling and emotion. The lillies of the field have a value beside their botanical one—a certain lightening of the heart accompanies the declaration that 'Solomon, in all his glory, was not arrayed like one of these.'

"The sound of the village bell, that comes mellowed from the valley to the traveller upon the hill, has a value beyond its accoustical one. The setting sun, when it mantles with the bloom of the rose the Alpine snows, has a value beyond its optical one. The starry heavens had for Imanuel Kant, as you know, a value beyond an astronomical one. Round about the intellect sweeps the horizon of emotions from which all our noblest impulses are derived. I think it very desirable to keep this horizon open; not to permit either priest or philosopher to draw down his shutters between you and it."

This topic, gentlemen, brings me to the statement of the object you have in coming here, as well as the functions of the university. On your part, you come to seek an education which shall answer to the character which I have described as necessary for you. On the part of my colleagues, I welcome you to a school whose aim is to offer you the opportunities for vigorous work, as well as the cultivation of the finer qualities of mind and heart, which shall prepare you to commence the labor which is to occupy the energies of your lifetime.

The character of the work before you is such as calls for careful and discriminating study.

I trust that you bring to it the same earnest spirit of inquiry which you manifest in other pursuits. It is the aim of your teachers not to instruct you simply in dry formularized knowledge, but to carry you with them and associate you in the energies of their own minds. You do not come to acquire a science and an art whose laws are already fully observed and defined, but you come as learners of, and searchers after, *truth*. Individual study and research is required of every one of you, for each has the opportunity to add something to the accumulated store of knowledge. Truly has it been said that "neglect of plain and patent truths has been the source of many of the great errors which appear on every side," and nothing, surely, contributes more largely to the maintenance of such errors than a dogmatic spirit of adherence to established formulas.

Careful and exact habits of study, combined with efficiency, are the essentials of success. Not every one, indeed, can fill the long furrows with seed, not every one can pile the broad fields with shocks of ripened grain; yet not a single hand is useless—that which cannot sow can weed, that which cannot reap can glean.

All are needed to complete the great work of growth and gathering in. The number of truly great men in the profession is comparatively small. It is not expected that *every one* shall become distinguished in some department of the profession, but each *can be exact*, and fitted to meet the requirements of the work falling in his sphere.

The object of the medical college is not to turn out, at each session, as large a number as possible of medical philosophers, but it is to educate the student in such a manner as best to prepare him for the daily work which he assumes in the practice of his profession.

To teach him *how* to observe, *what* to observe, and how to *classify* and apply the results of his observation. There is nothing in such an education to prevent one from aspiring to the heights of science or of philosophy, but rather to encourage and aid him by laying such a foundntion as shall support the fairest superstructure which patient toil can raise.

Beyond the immediate stimulus of future success, lie attractions in the study of the profession which you have chosen, surpassing in interest those of any other pursuit.

The intricacies of anatomy, the wonderful vital processes of physiology, organic chemistry, the therapeutic and physiological action of medicines, the science and art of surgery, the study of disease and abnormal conditions, all offer an endless field of observation, and one which deepens in its attractions as you advance within it. Yet these subjects form but a part of the curriculum of study placed before you. The advances in knowledge during the past half century place you to-day above the students of the last generation, and the steady accumulations of knowledge which are fostered and preserved by our civilization, will in future times give a power and force of character to our profession which will surpass the brilliant position of to-day. You are studying with constantly increasing advantages. Your opportunities are to-day greater than those even of your immediate predecessors, and you ought consequently to surpass them in your attainments.

I have urged upon you the necessity of a recognition of the scientific character of your profession, yet I would remind you that to prove available you must conjoin with it that which shall make it practical. Science, unless it can be made useful, would be only an amusement, or recreation. To be of value it must be joined with art. In reviewing the social history of man, we recognize medicine as commencing as a simple art. As we advance from the savage state to that of civilization, the steady development of the art toward the dignity of a science is apparent, until to-day, the union of its two phases, the art and the science, give it the eleva'ed position which it occupies.

While, then, your earnest aim should be to master as fully as possible the science of medicine, you must as earnestly cultivate the art, in order to make your scientific attainments of real and practical value to you.

It is this happy combination which often is the explanation of the differing success of equally cultivated men, in our profession.

It is the aim of the clinical teachers of this institution, to instruct you in the art of applying your science, and in cultivating that *tact* in the application of your science to practice, which shall make you masters of your art. The medical *philosopher* may, perhaps, rest contented with his science; but the practitioner must ever labor to sustain a constant and coequal development of both the science and the art.

Gentlemen, your work is before you. You have seen the relations of medicine to the past; you know the relations of medicine to the present; you can look hopefully forward to its relations in the future.

Let me, in concluding, express the hope that, for you the future may prove the unfolding and expanding of the germs of knowledge which have started here. That you will feel that, upon each one of you, it is incumbent to prove yourselves men of thought, men of knowledge, and above all *scholars*. Youth is no bar to entrance upon the cause. The scholar of maturer years may have the largest knowledge, the strictest training. On none so much as on him do the interests of learning depend for prudence and retrospection. But where hopefulness is needed, where vigor is required, we may be content to trust to young spirits. The *union* of the young and the old can alone achieve the scholars march.

It is for the young to throw forward the glances which give life to the array, while its security is insured by the backward looks of the old. The mountain tops behind lost sight of, the way becomes uncertain; it is weary if the peaks before are not discerned.

Enlist, then, in the cause which calls you, the cause which depends upon you, Shape your studies according to the true ideal, and you will find them crowned with beauty and happiness, with success without and blessings within.

